# Effect of Acupoint Embedding on Serum Leptin and Hypothalamus Leptin Receptor Expression in Rats with Simple Obesity

**DOI:** 10.1155/2021/3500409

**Published:** 2021-10-06

**Authors:** Qiming Yang, Liwei Xing, Qinzuo Dong, Hongxi Chen, Chunlin Xia, Xianwu Yue, Yuanfeng Wang, Li Li, Yingjing Luo, Mingxing Lai, Dong Zhao, Zhiyan Zhang, Rong Zhao

**Affiliations:** ^1^Yunnan University of Traditional Chinese Medicine, Kunming Hospital of Traditional Chinese Medicine, Kunming, China; ^2^Yunnan University of Traditional Chinese Medicine, Kunming, China; ^3^Affiliated Hospital of Yunnan University, Kunming, China; ^4^Kunming Health Commission, Kunming, China

## Abstract

**Background:**

Acupoint embedding treatment on obesity has been applied in clinical practice for many years and has achieved obvious efficacy. However, animal experimental studies on acupoint embedding are relatively few, and its mechanism remains unclear.

**Methods:**

We established a simple obese rat model using a high-fat diet for 8 weeks. Acupoint embedding therapy was performed once a week for 4 weeks. After the treatment, serum leptin, triglyceride (TG), cholesterol (TC), low-density lipoprotein (LDL), and high-density lipoprotein (HDL) levels were detected by radioimmunoassay, HE staining was used for fat morphology analysis, and immunohistochemical method was used to detect the expression of leptin receptor in hypothalamus.

**Results:**

Compared with model group, acupoint embedding treatment can reduce body weight and Lee's index, reduce serum leptin, TG, TC, and LDL level, increase HDL level, change the morphology and number of adipocytes, and increase the expression of leptin receptor in hypothalamus.

**Conclusion:**

Acupoint embedding therapy can reduce the level of leptin in blood, increase the number of leptin receptors in hypothalamus, enhance the biological effect of leptin, alleviate the leptin resistance in obese body, change the shape of fat, and regulate the level of blood lipid, so as to achieve the goal of weight loss.

## 1. Introduction

Obesity is a chronic disease characterized by excessive amount of fat in the body, increased volume of fat cells, and abnormal distribution. It is a recognized risk factor or pathogenic factor of a variety of chronic diseases in modern society. The latest authoritative figures put the proportion of the population as high as 12 percent as obese [1], and obesity has become one of the most serious health problems in the 21st century [2]. Obesity not only affects physical appearance, but also increases the incidence of cardiovascular and cerebrovascular diseases, diabetes, hyperlipidemia, infertility, and tumors, which seriously affects human physical and mental health and weakens body resistance [3].

The most fundamental cause of obesity is that energy intake is greater than consumption, and excess energy is stored in the body in the form of fat, resulting in excessive accumulation of body fat [4]; that is, the imbalance between energy storage and consumption is the basis of obesity [5] Leptin is one of the most important energy regulation signals, which is not only present in the blood, but also widely distributed in the brain tissue and gastrointestinal tract. It can suppress appetite, reduce energy intake, and increase energy consumption, and it plays an important role in inhibiting fat synthesis [6]. When leptin binds to hypothalamic receptor (OB-R), it alters the expression of specific neuropeptides produced by other genes in hypothalamic neurons, thereby regulating the body's energy balance [7]. However, clinical studies have found that the leptin level of obese patients is generally higher than that of people with normal weight, which is positively correlated with BMI [8]. Hyperleptinemia in obese patients does not exert the function of leptin as an appetite suppressant, and this phenomenon is considered to be leptin resistance. Some scholars believe that the mechanism of leptin resistance is the result of inhibition on multiple levels of leptin signal transduction pathway, including changes in leptin receptor expression, blocking of leptin receptor postsignaling pathway, or inhibition of downstream signaling factors [9]. So leptin and its receptors play a central role in weight regulation, and any disruption of this signaling system can lead to obesity.

Acupoint embedding treatment on obesity has been applied in clinical practice in recent years and has achieved obvious curative effects. Acupoint embedding therapy is a continuation of acupuncture, which embeds biological protein lines that can be absorbed by the body into the acupoints and stimulates the acupoints continuously through the protein lines for a long time to prevent and cure diseases. Compared with ordinary acupuncture, acupoint embedding has the advantages of longer duration of effect, fewer visits, and good long-term efficacy [10], which improves the quality of life of patients and saves medical costs. However, animal experiments on acupoint embedding are relatively few, and its mechanism is still unclear.

In this study, we established a simple obesity rat model by using a high-fat diet for 8 weeks and used this model to explore the mechanism of acupoint embedding on simple obesity.

## 2. Materials and Methods

### 2.1. Animals

Sprague Dawley (SD) female rats (150–180 g) were purchased from Hunan Slike Jingda Experimental Animal Co., Ltd., license number: SCXK (Hunan) 2016-0002. The study was conducted in accordance with the local code of ethics for animal care and use. According to Meng et al. [11], rats in the high-fat group were fed with high-fat diet (Table 1) for 8 weeks of continuous feeding. The criteria for successful modeling of obese rats were as follows: the body weight of obese rats was 20% higher than that of normal rats. Rats were randomly divided into 3 groups, 10 in each group: normal control group: normal feeding without intervention measures; model control group: no intervention measures, high-fat feed feeding; acupoint embedding group: high-fat feed feeding, with acupoint embedding.

### 2.2. Acupoint Embedding Operation

**Figure 1:**
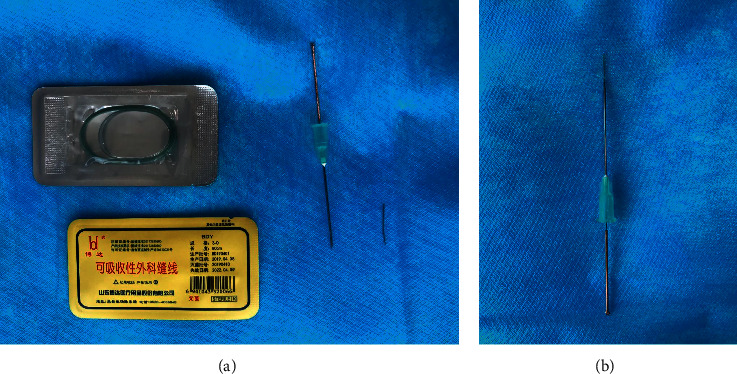
Structure of embedding needle. Cut the medical absorbable thread into 3 mm equal length line segments (a), and insert the thread body through one side of the embedding needle, and the other side was inserted by the flat-head needle (b).

According to the orientation of animal acupuncture points commonly used in 《Experiment Guidance and Skill Training of Experimental Acupuncture and Moxibustion》 [12], before embedding the thread, put the medical absorbable thread (JinHuan medical gut, 3-0) on the disinfectant bending plate, cut it into 3 mm equal length segments with the disinfectant thread shears, and put the thread body into the tip of the embedding needle (No.8 disposable injection needle, 0.8 × 38 TWLB, OuJian disposable sterile injection needle). A flat-head needle (Shunhe flat-head acupuncture needle, size 0.35 × 35 mm) was inserted into the pinhole on the other side to form a thread embedding needle (Figure 1). After disinfecting the skin at the acupoints of rats, we selected “Zhongwan,” “Tianshu,” and “Hou Sanli” acupoints for oblique pruning. According to the study of Tekus E [13], the average thickness of subcutaneous fat layer in rats can be more than 5 mm, so 4 mm was selected as the predetermined depth of embedding wire to ensure that the embedding wire reaches the fat layer. We slowly pulled out the needle while injecting the thread into the acupoints. The absorbable line was buried in the acupoints of rats after observing whether the gut was exposed outside the body. Acupoint embedding therapy was performed once a week for 4 consecutive weeks. All rats were sacrificed immediately after treatment.

### 2.3. Body Weight and Lee's Index

Body weight and body length of all rats were measured every 7 days during modeling and intervention, using an electronic balance with an accuracy of 0.5 g. Lee's index = 3√ (weight × 1000)/body length. Note: Lay the rats on the table with the back facing up to straighten the body. Place cardboard barriers around the rats to restrict their activities and keep them relatively still. The length from the tip of the nose to the anus is the length of the body, and it is measured with a ruler.

### 2.4. Biochemical and Hematological Analysis

After treatment, rats in each group were fasted overnight, blood samples were taken from abdominal aorta the next day and centrifuged at 2000 r/min for 20 min, serum separation was followed by immunoassay for leptin, as previously reported [13], and the kit of Cloud-Clone Corp. (CCC, USA) was used. Triglyceride (TG), cholesterol (TC), low-density lipoprotein (LDL), and high-density lipoprotein (HDL) were detected by ChemRay 240 and ChemRay 800 automatic biochemical analyzer (Shenzhen Redu Life Science and Technology).

### 2.5. Measurement of Fat Coefficient (White Fat and Brown Fat)

After the treatment, the rats were sacrificed. The white fat around the abdomen, genitalia, and the kidney and the brown fat around the shoulder were separated from each group. The wet weight of the fat was measured by an electronic balance (accuracy was 0.1 mg) after filtration with a filter paper and recorded.

### 2.6. Morphological Evaluation by HE Staining

Adipose tissue with a thickness of 0.2–0.3 cm and a size of 1.5 cm ∗ 1.5 cm ∗ 0.3 cm in the same part of the rats' groin in each group was selected and immersed in 4% paraformaldehyde solution for 48 h. After that, the adipose tissue was dehydrated with conventional gradient alcohol, xylene was transparent, and it was paraffin embedded. Routine sectioning was done on a microslicer. The thickness of the slices was controlled at about 5 *μ*m, the slices were spread, baked (baked at 60°C for 3 hours), dewaxed, and hydrated by xylene step by step and then stained with hematoxylin-eosin (HE), and the slices were sealed with gum, as reported by Li et al. [14]. Ten fields were randomly selected under a 400x light microscope, and the number of adipocytes in the whole field was recorded. The size of adipocytes in each group was measured and compared, and the average value of each case was taken.

### 2.7. Detection of Expression of Leptin Receptor by Immunohistochemistry

After blood collection, the rats were fixed and perfused to extract the hypothalamus. The samples were quickly placed into a fixed solution containing 0.2 kg/mol PBS of 4% paraformaldehyde containing 1/1000 DEPC, dehydrated with gradient alcohol at room temperature, transparent with xylene, embedded in paraffin after paraffin immersion for 30 min, and sected (the thickness was 5 *μ*m). Next, the following procedures were performed to complete the operation: (1) The tissue sections were hydrated and dewaxed. ② 3% H_2_O_2_ deionization was used to incubate it for five to ten minutes to block the peroxidase in it, and distilled water was used to wash it for three times. (3) The heat repair antigen was carried out by microwave, repeated twice, and then cooled and washed. (4) Rat awntibody drops were added to the prepared working solution overnight. (5) IgG (biotin sheep anti-rabbit) was added, and 0.1 kg/mol PBS was used for repeated washing for three times. ⑥ DAB was used for color rendering. ⑦ Hematoxylin was used for redyeing, sealing, transparency, and dehydration. (8) After observing and taking pictures, CMIAS image analysis system was used to conduct directional analysis of cell count. Immune positive cells were yellow brown cells, as reported by Yang et al. [15]. In each group, five slices of rat hypothalamus with a field of vision of more than 40 times were selected.

### 2.8. Statistical Analysis

In this experiment, SPSS25.0 statistical software was used for data sorting, screening, and statistical analysis. Measurement data were recorded in $\bar{x}\pm s$ form when obeying normal distribution. Paired *t*-test was used for intragroup comparison before and after treatment, and analysis of variance was used for intergroup comparison. The test level of 0.05 was used, and *P* < 0.05 indicated that the difference was statistically significant.

## 3. Results

### 3.1. Effects of Acupoint Embedding on Body Weight and Lee's Index

**Figure 2:**
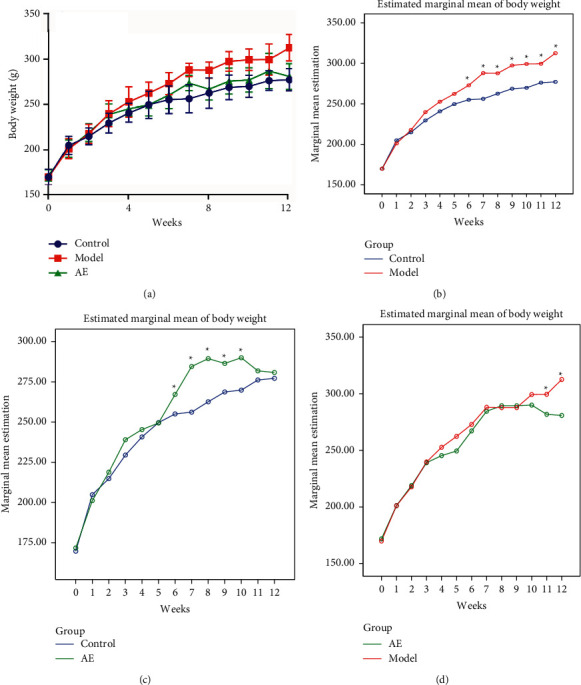
Effect of acupoint embedding on body weight of 12W rats. Control: normal control group. Model: model control group. AE: acupoint embedding group.  ^*∗*^*P* < 0.05. The general trend of weight change in each group (a). Line chart of simple effect analysis on body weight of control group and model group (b). Line chart of simple effect analysis on body weight of control group and AE group (c). Line chart of simple effect analysis on body weight of model group and AE group (d).

**Figure 3:**
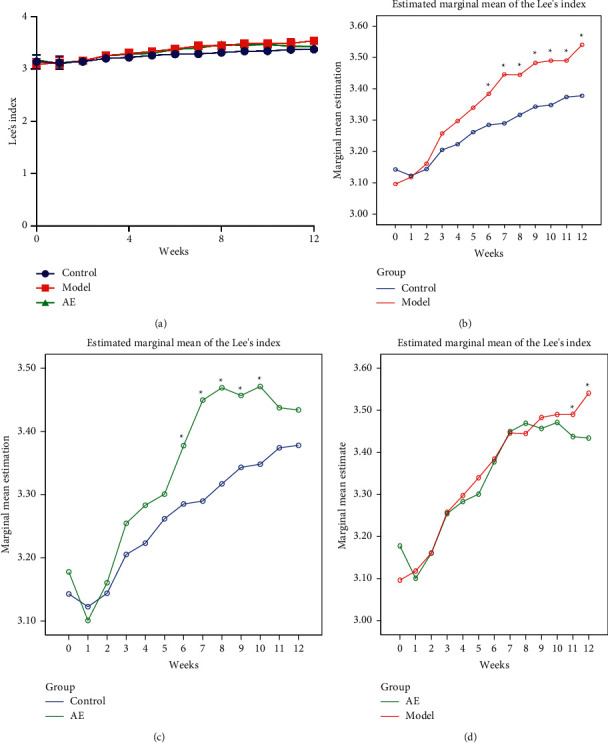
Effect of acupoint embedding on Lee's index of 12W rats. Control: normal control group. Model: model control group. AE: acupoint embedding group. ^*∗*^*P* < 0.05. The general trend of Lee's index change in each group (a). Line chart of simple effect analysis on Lee's index of control group and model group (b). Line chart of simple effect analysis on Lee's index of control group and AE group (c). Line chart of simple effect analysis on Lee's index of model group and AE group (d).

Changes in body weight and Lee's index of the rats during the 12-week experiment are shown in Figures 2 and 3. The general trend of body weight and Lee's index among each group were shown in Figures 2(a) and 3(a). Two-way repeated ANOVA and post hoc test were conducted for experimental rigor. Time and group interaction effects of body weight and Lee's index between control and model group, control and AE group, and model and AE group were significant (*P* < 0.05). After simple effect analysis, the simple effect analysis line chart of body weight and Lee's index between each group were shown in Figures 2(b)–2(d), 3(b)–3(d), respectively. In the first 7 weeks, the body weight and Lee's index in each group increased steadily. From the 8th week, the body weight and Lee's index in the two high-fat diet groups were significantly higher than those fed normal diet. And the body weight in the model group was 20% higher than that in the control group, with statistically significant differences (*P* < 0.01), indicating that the modeling was successful. From the 9th week (start of the acupoint embedding intervention) to the 12th week (end of the experiment), the average body weight and Lee's index of the model group were significantly increased compared with the control group, and the difference was significant (*P* < 0.05); see Figures 2(b) and 3(b). From the 11th week, the average body weight and Lee's index of acupoint embedding group increased slowly, which had significant difference compared with the model group (*P* < 0.05); see Figures 2(d) and 3(d). But they had no significant difference compared with the control group (*P* > 0.05); see Figures 2(c) and 3(c). Compared with the model group, acupoint embedding can reduce body weight and Lee's index.

### 3.2. Effects of Acupoint Embedding on Serum Leptin

As shown in Table 2, compared with the control group, the serum leptin level of model group rats was significantly increased (*P* < 0.05), whereas the serum leptin level in acupoint embedding group was significantly decreased after treatment compared with model group (*P* < 0.05). These results suggested that obesity may be related to the increase of leptin level and the failure of leptin to exert its normal biological effect, which is consistent with previous studies [16]. Acupoint embedding therapy can reduce serum leptin level.

### 3.3. Effects of Acupoint Embedding on Blood Lipids

As shown in Table 2, the levels of serum TG, TC, and LDL were significantly increased in the model group relative to the controls, whereas the effects were blocked by acupoint embedding treatment (*P* < 0.05). The levels of serum HDL were significantly decreased in the model group relative to the controls, and the effects were blocked by acupoint embedding treatment (*P* < 0.05). The above results suggested that simple obesity rats may have abnormal lipid metabolism and lipids. Acupoint embedding therapy can regulate the level of blood lipid and alleviate dyslipidemia.

### 3.4. Effects of Acupoint Embedding on Fat Coefficient

As shown in Table 3, the white fat coefficient and brown fat coefficient were both increased in the model group relative to the controls (*P* < 0.05). And the white fat coefficient and brown fat coefficient of acupoint embedding group decreased after treatment compared with the model group (*P* < 0.05), but there was no difference compared with the control group (*P* > 0.05). The above results suggested that the white fat and brown fat in obese rats were increased compared with control rats, and the white fat and brown fat in obese rats were decreased after acupoint embedding treatment.

### 3.5. Effects of Acupoint Embedding on Adipose Morphology

**Figure 4:**
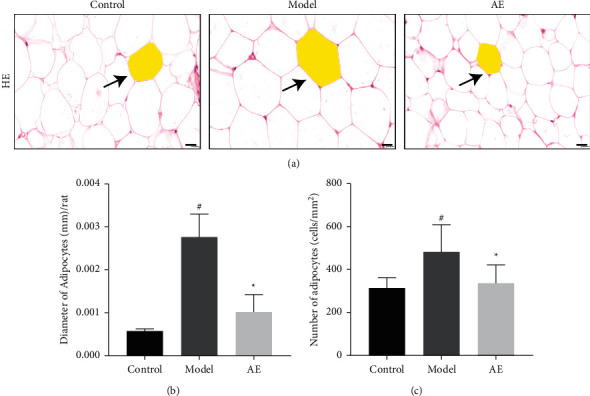
Effects of acupoint embedding on adipose morphology. At least 3 400-fold visual fields were randomly selected from each section of each group for photographing (a). When photographing, try to fill the entire field of view with tissue to ensure the same background light for each photo. Using Image-Pro Plus 6.0 software and 400x scale as the standard, an appropriate field of vision was selected. Diameters of 5 adipocytes (mm) were measured for each section, and the average value was calculated (c). The visual field area of each section was measured and analyzed (mm^2^), the number of adipocytes in the visual field was counted (number), and the density of adipocytes per unit area (number/mm^2^) = the number of adipocytes/visual field area (mm^2^) which was calculated (b). ^#^Compared with normal control group, *P* < 0.05; ^*∗*^*P* < 0.05 compared with model control group.

As shown in Figure 4(a), the morphology of groin adipocytes of rats in each group stained with HE was observed under the field of 400x light microscope. In the adipocytes of the control group, the volume of adipocytes was generally large, and the cell margins were irregular and scattered. In the adipose tissue of model group, adipose cells were large, round, and compact. In the adipose tissue of acupoint embedding group, the adipose cells were small, uniform, and full.

As shown in Figure 4(b), compared with the control group, the adipocyte count of model group rats was significantly different (*P* < 0.05). Compared with the obesity control group, the adipocyte count in acupoint embedding group was significantly different after treatment (*P* < 0.05). Compared with the control group, the adipocyte count in acupoint embedding group had no statistical difference (*P* > 0.05).

As shown in Figure 4(c), compared with the control group, the size of adipocytes in the model group was significantly different (*P* < 0.05). Compared with the model group, the size of adipocytes in acupoint embedding group was significantly different after treatment (*P* < 0.05). Compared with the control group, there was no significant difference in the size of adipocytes in acupoint embedding group (*P* > 0.05).

The comparison of adipocyte morphology between the model group and the acupoint embedding group showed that, after the treatment, the adipocytes in the acupoint embedding group showed an increasing trend, while the cell morphology showed a decreasing trend, suggesting that the treatment could change the morphology and number of adipocytes.

### 3.6. Effects of Acupoint Embedding on Leptin Receptor Expression in Hypothalamus

**Figure 5:**
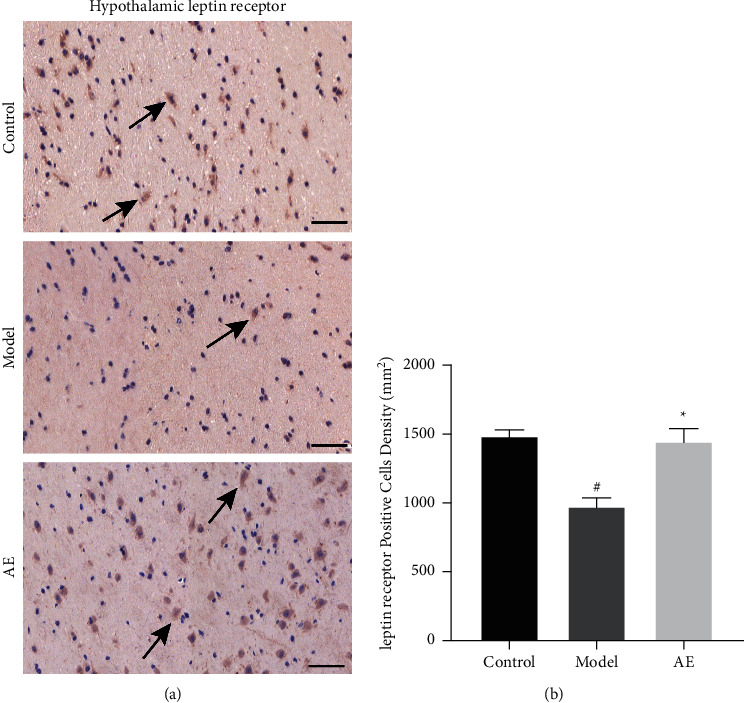
Effects of acupoint embedding on leptin receptor expression in hypothalamus. At least three 400-fold fields were randomly selected from the lateral hypothalamic region of each rat in each group for photography (a). CMIAS image analysis system was used to conduct directional analysis on the number of cells, and the number of weak and strong positive cells in the measured area was analyzed and calculated. The immune positive cells were yellow brown cells. Leptin receptor-positive cell density in hypothalamus: number of positive cells/tissue area to be measured (b). ^#^Compared with normal control group, *P* < 0.05; ^*∗*^*P* < 0.05 compared with model control group.

The immunohistochemical results were shown in Figure 5(a). As shown in Figure 5(b), compared with the control group, the expression of leptin receptor in hypothalamus of obese control group rats was significantly decreased (*P* < 0.05). Combined with the above significant increase of serum leptin level in obese control rats, it suggests that there may be leptin resistance in obese rats. But after 4 weeks of treatment, the expression of leptin receptor in hypothalamus of acupoint embedding group was significantly increased compared with the model group (*P* < 0.05), and there was no difference compared with the control group (*P* > 0.05). These results suggest that the leptin resistance can be reversed by acupoint catgut embedding therapy, which can improve the expression of leptin receptor in hypothalamus and reduce the level of leptin in blood.

## 4. Discussion

The latest epidemiological data suggests that, by the end of 2015, about 800 million people which means 17 percent of the population were obese [17]. There are about 4 million deaths each year as a direct result of high BMI, accounting for about 7.1 percent of all deaths [18]. At present, the medicaments that treat obesity use methamphetamine, amino acid mostly which are with strong side effects. Other treatments focus on lifestyle changes such as diet and physical activity [19]. It is urgent to find effective and less side-effect treatment methods due to the great harm and insufficient treatment methods of simple obesity. In recent years, TCM therapy has achieved remarkable curative effect in treating simple obesity. Among them, the acupoint embedding method is the synthesis and development of the acupuncture and embedding needle. It produces immediate and lasting effects on human body, makes up for the short stimulation time of acupuncture, and reduces the number of times of patients visiting a doctor. It is especially suitable for modern busy obese people [20]. The latest meta-analysis shows that acupoint embedding has a significant effect on simple obesity with less incidence of adverse reactions [21]. Qi and Cheng [22] showed that, after acupoint catgut embedding treatment, 32 of the 60 female patients were significantly effective, 22 were effective, and the effective rate was as high as 90%. Tao et al. [23] used acupoint embedding therapy combined with traditional Chinese medicine to treat obese PCOS patients. After treatment, the levels of BMI and adipocytokine TNF-*α* in patients in the acupuncture group were significantly reduced.

As a product secreted by adipocytes, leptin is a peptide hormone that acts on many tissues and organs through its receptors. Zhang et al. [24] successfully cloned the obesity-related gene in mice and identified the corresponding obesity-related gene and its protein product, leptin. It is a circulating hormone secreted by adipocytes, and it is currently believed that leptin can regulate fat deposition in the body through three ways [25]: (1) regulation of energy balance through several brain mechanisms, such as decreased appetite, decreased energy intake, and increased energy expenditure; (2) promoting the expression of uncoupled protein mRNA in brown adipose tissue, which can improve the heat production, energy consumption, and metabolic rate; (3) inhibit fat synthesis. It acts on the weight regulation center of the hypothalamus, thereby causing appetite reduction, increasing energy consumption, and finally reducing weight. Leptin resistance (LR) refers to the insensitivity or unresponsiveness of tissue to the regulation of leptin. Most obese people have hyperleptinemia [26], and the possible mechanism of obesity LR is as follows: (1) LP deficiency, hyperleptinemia in 5% of obese patients; (2) LP receptor mutation. LP receptor defect cannot accept LP information [27]; (3) LP transport disorder. LEP enters central nervous system through the blood-brain barrier. (4) LP signal inhibition, LP receptor postsignal transduction disorder [28]. In this study, we found that leptin levels were significantly increased, but hypothalamus leptin receptor expression decreased in high-fat induced obese rats, indicating there exists leptin resistance in the obese model group. Endogenous leptin is not effective in increasing energy expenditure, reducing food intake, and regulating energy balance which is consistent with previous research. After treatment, the serum leptin in acupoint embedding group decreased, and the difference was statistically significant compared with the model group (*P* < 0.05), indicating that acupuncture can effectively reduce the excessive leptin in the blood of obese rats and alleviate the leptin resistance.

Leptin receptor (OB-R) is widely distributed in the hypothalamus [29]. The combination of leptin and OB-R in the hypothalamus changes the expression of specific neuropeptides produced by other genes in the hypothalamus neurons, thus regulating the balance of energy in the body. Leptin is secreted by surrounding adipose tissue, and its levels reflect fat storage. It reaches brain tissue through blood circulation, binds to leptin receptors that feed on the central nervous system, and sends signals to the central nervous system, producing a series of effects: Inhibitory neuropeptide Y (NPY) promotes the combination of melanocorticoid and melanocorticoid receptor 4 and produces the completely opposite effect to S protein, so as to inhibit feeding and the occurrence of obesity, increase the expression of uncoupling protein in adipose tissue, and consume excess energy in the form of heat energy [7]. Leptin and leptin receptors therefore play a central role in weight regulation, and any disruption of this signaling system can lead to obesity. In this study, immunohistochemical analysis of the hypothalamic leptin receptor in obese rats induced by high-fat diet was conducted, and it was found that the expression of the hypothalamic leptin receptor was significantly decreased in the obesity model group, indicating that the leptin resistance in obese rats may be caused by the lack of receptors in the central part and the decreased expression of the hypothalamic receptor. After treatment, the expression of leptin receptor in hypothalamus was significantly increased in acupoint embedding group, indicating that acupoint embedding therapy can significantly increase the number of leptin receptor in hypothalamus, enhance the biological effect of leptin, and alleviate the leptin resistance in obese bodies, so as to achieve the goal of weight loss.

Previous studies have shown that increased adipocyte volume is an important histological feature of obesity [30, 31]. The results of this study showed that the diameter, area, and volume of adipocytes in the model group were increased compared with those in the normal group (*P* < 0.05), and the body weight and Lee's index were also significantly increased. This phenomenon suggested that obese rats in the model group had the characteristics of increased adipocyte volume and significantly increased lipid storage capacity, which was consistent with the histological changes of human obesity. Compared with the model group, the diameter of adipocytes in acupoint embedding group was significantly decreased (*P* < 0.05), and the body weight and Lee's index were also significantly decreased, which was consistent with the appearance of flat and round adipocytes distributed in sheet shape. This phenomenon can reflect the decrease of the storage capacity of adipose cells, indicating that the structure of adipose tissue changes after the overall adjustment of fat metabolism after acupoint embedding. It should be noted that the results of this study showed that, after acupoint embedding treatment, the number of adipocytes in the acupoint embedding group was higher than that in the normal control group or model control group in the field of vision (*P* < 0.05), which was inconsistent with previous studies [32]. This phenomenon was considered to be related to the level of embedding at the acupoints. Li et al. [14] conducted relevant studies, and the results showed that acupoint catgut embedding at the fat layer, muscle layer, and mixed layer had different influences on the count of adipocytes. This study aimed to study the mechanism of acupoint embedding at the subcutaneous fat layer, which was consistent with the clinical level of acupoint embedding, so embedding at the fat layer was selected.

There are two types of fat in the human body, brown adipose tissue and white adipose tissue, which play opposite roles in energy metabolism. The function of WAT is mainly to store the excess energy in the body in the form of fat, while BAT is mainly to emit the energy generated by lipid oxidation in the form of heat through the uncoupling of its mitochondria, thus playing its role [33]. This study suggested that the white fat coefficient and brown fat coefficient of obese rats were higher than those of normal rats, and the trend of decrease after acupoint embedding treatment indicated that acupoint embedding could reduce the white and brown fat coefficient of obese rats, so as to play the role of energy metabolism of fat. Obesity is the result of the imbalance of energy metabolism, which will inevitably lead to the disorder of lipid metabolism. This study showed that, compared with normal rats, the high-fat diet induced the increase of lipid level in obese rats (*P* < 0.05). After acupoint embedding treatment, with the weight reduction of obese rats, TC, TG, and LDL significantly decreased, and HDL-C increased, indicating that acupoint embedding can alleviate lipid while reducing body weight. It can reduce weight and adjust lipid metabolism and reduce the incidence of cardiovascular and cerebrovascular diseases. Acupoint embedding reduces the level of blood lipid and changes the fat structure which is an important part of weight loss. It is also the result of energy metabolism and fat metabolism tending to be normal.

In conclusion, our results show that, in leptin resistance, reduction of hypothalamic leptin receptor expression exists in the obese rats induced by high-fat diet; this makes the leptin and leptin receptor cannot effectively play the role of energy balance regulation and cause the change of the adipose cell morphology and secondary elevated blood lipid levels and lipid metabolism disorders. This process is similar to the deposition of fat and food in traditional Chinese medicine, which cannot be absorbed by the body, that is, the pathogenesis of “dysfunction of spleen in transportation” in connotation and correlation. Acupoint embedding therapy can reverse this process, reduce the level of leptin in blood, increase the number of leptin receptors in hypothalamus, enhance the biological effect of leptin, alleviate the leptin resistance in obese body, change the shape of fat, and regulate the level of blood lipid, so as to achieve the goal of weight loss. Further research is needed to determine the molecular mechanism of acupoint embedding.

## Figures and Tables

**Table 1:** Rat chow composition (%).

Ingredients	Standard diet (%)	High-fat diet
Barley powder	20	10%
Bran	16	8%
Corn flour	16	8%
Flour	10	10%
Fish meal	10	5%
Bone meal	5	2.5%
Salt	2	3%
Yeast	1	0.5%
Alfalfa meal	20	10%
Lard	—	12%
Milk powder	—	10%
Egg yolk powder	—	5%
Casein	—	5%
Peanut	—	5%
Sucrose	—	5%
Sesame oil	—	1%
Vitamin A + D	—	10 drops

**Table 2:** Comparison of fat coefficient of each group after treatment ($\bar{x}$ ± *s*).

	Normal control group	Model control group	Acupoint embedding group
WAT	0.0213 ± 0.0078	0.0301 ± 0.0045^*∗*^	0.0207 ± 0.0062^#^
BAT	0.0016 ± 0.0005	0.0031 ± 0.0006^*∗*^	0.0020 ± 0.004^#^

*Note.* ^*∗*^ *P* < 0.05 compared with normal control group; ^#^*P* < 0.05 compared with model control group.

**Table 3:** Blood lipid indexes after treatment.

Items	Normal control group	Model control group	Acupoint embedding group
Leptin	0.42 ± 0.25	5.82 ± 1.65^*∗*^	0.82 ± 0.37^#^
TG	0.171 ± 0.180	0.679 ± 0.098^*∗*^	0.188 ± 0.006^#^
TC	1.114 ± 0.093	2.859 ± 0.072^*∗*^	1.594 ± 0.074^#^
HDL	0.930 ± 0.077	0.543 ± 0.089^*∗*^	0.883 ± 0.037^#^
LDL	0.180 ± 0.033	0.267 ± 0.031^*∗*^	0.203 ± 0.006^#^

*Note.* ^*∗*^ *P* < 0.05 compared with normal control group; ^#^*P* < 0.05 compared with model control group.

## Data Availability

The data used to support the findings of this study are available from the corresponding author upon request.

## References

[1] Low S., Chin M. C., Deurenberg-Yap M. (2009). Review on epidemic of obesity. *Annals Academy of Medicine Singapore*.

[2] Must A., Strauss R. S. (1999). Risks and consequences of childhood and adolescent obesity. *International Journal of Obesity*.

[3] Zou D. J. (1999). *Clinical Application of Clinical Obesity*.

[4] Hill J. O., Wyatt H. R., Peters J. C. (2012). Energy balance and obesity. *Circulation*.

[5] Wang Z., Cao Y. (2011). Brown adipose tissue metabolism and obesity. *Life Science Research*.

[6] Zhang Y., Chua S. (2017). Leptin function and regulation. *Comprehensive Physiology*.

[7] Wang S. (2003). Leptin resistance and obesity. *Foreign Journal of Medical Hygiene*.

[8] El-Haggar S. M., Mostafa T. M. (2015). Adipokines and biochemical changes in Egyptian obese subjects: possible variation with sex and degree of obesity. *Endocrine*.

[9] Crujeiras A. B., Carreira M. C., Cabia B., Andrade S., Amil M., Casanueva F. F. (2015). Leptin resistance in obesity: an epigenetic landscape. *Life Sciences*.

[10] Yan R. H., Bai J., Zhang Y., Gu J. S., Yu J. (2010). The long-term effect of acupoint catembedding therapy on simple obesity. *China Aesthetic Medicine*.

[11] Meng X. R., Zhu X. S., Yang C. Y., Jing A., Tan Q. W., Yu H. J. (2019). Effect of catgut embedding at tianshu acupoint on insulin resistance in obese rats. *Clinical journal of acupuncture and moxibustion*.

[12] Li Z. R. (2007). Experimental guidance and skill training of experimental acupuncture and moxibustion. *China Press of Traditional Chinese Medicine*.

[13] Wu J., Zhang H., Zheng H., Jiang Y. (2014). Hepatic inflammation scores correlate with common carotid intima-media thickness in rats with NAFLD induced by a high-fat diet. *BMC Veterinary Research*.

[14] Li X. Y., Liu Z. D., Zhao C., Chen C. L., Liang W. (2017). Effects of acupoint emplantation on obesity and lipid metabolism in rats with simple obesity. *Journal of Clinical Acupuncture and Moxibustion*.

[15] Yang C. Z., Ma Y., Xu Y. L., Wang Y., Wang Y., Zhang D. W. (2007). Effects of acupuncture on serum leptin content and hypothalamus leptin receptor expression in simple obesity rats. *Acupuncture Research*.

[16] Fei H., Okano H. J., Li C. (1997). Anatomic localization of alternatively spliced leptin receptors (Ob-R) in mouse brain and other tissues. *Proceedings of the National Academy of Sciences*.

[17] The GBD 2015 Obesity Collaborators (2017). Health effects of overweight and obesity in 195 countries over 25Years. *New England Journal of Medicine*.

[18] Committee on Quality Measures for the Healthy People Leading Health Indicators, Practice H., Medicine I. O. (2013). *Toward Quality Measures for Population Health and the Leading Health Indicators*.

[19] Shen H. L., Zhang Y. (2018). Research status of diet and drug treatment for obesity. *Medical Review*.

[20] Hu X. Y., Gao Y. L., Wang Y., Hu Z. H., Shi Y. (2021). Meta-analysis of acupoint catgut embedding in treatment of simple obesity. *Henan Journal of Traditional Chinese Medicine*.

[21] Liao J. Q., Song X., Chen Y., Liang L. C., Wang S. X. (2014). Meta-analysis of randomized controlled clinical studies on acupoint catgut embedding in the treatment of simple obesity. *Chinese Acupuncture & Moxibustion*.

[22] Qi F. J., Cheng J. J. (2006). Clinical observation of 60 cases of weight loss by acupoint embedding thread. *Journal of Acupuncture and Moxibustion*.

[23] Tao L. L., Fu Y. H., Xie P. P. (2009). Effects of acupoint catgut emburying combined with spleen and expectorant Chinese medicine on insulin resistance and adipocytokines in obese polycystic ovary syndrome. *Journal of Guangzhou University of Traditional Chinese Medicine*.

[24] Zhang Y., Proenca R., Maffei M., Barone M., Leopold L., Friedman J. M. (1994). Positional cloning of the mouse obese gene and its human homologue. *Nature*.

[25] He Y. X. (1999). Advances in molecular biology of the etiology of obesity. *Journal of Tianjin Institute of Physical Education*.

[26] Havel P. J., Kasim-Karakas S., Mueller W., Johnson P. R., Gingerich R. L., Stern J. S. (1996). Relationship of plasma leptin to plasma insulin and adiposity in normal weight and overweight women: effects of dietary fat content and sustained weight loss. *Journal of Clinical Endocrinology & Metabolism*.

[27] Chung W. K., Power-Kehoe L., Chua M. (1997). Exonic and intronic sequence variation in the human leptin receptor gene (LEPR). *Diabetes*.

[28] Caro J. F., Kolaczynski J. W., Nyce M. R. (1996). Decreased cerebrospinal-fluid/serum leptin ratio in obesity: a possible mechanism for leptin resistance. *The Lancet*.

[29] Kalra S. P., Dube M. G., Pu S., Xu B., Horvath T. L., Kalra P. S. (1999). Interacting appetite-regulating pathways in the hypothalamic regulation of body weight. *Endocrine Reviews*.

[30] Wang S. J., Li Q., She Y. F., Li A. Y., Xu H. Z., Zhao Z. G. (2005). Effects of electroacupuncture on lipid metabolism in obese rats induced by sodium glutamate. *Chinese Acupuncture*.

[31] Liu X., Qu Y. T., He J. F. (2015). Anti-obesity effect of electroacupuncture on diet-induced simple obesity in rats and its mechanism. *Journal of Beijing University of Traditional Chinese Medicine*.

[32] Yan R. H. (2013). *Clinical Efficacy and Molecular Mechanism of Acupoint Catgut Embedding in the Treatment of Simple Obesity*.

[33] Farmer S. R. (2008). Molecular determinants of brown adipocyte formation and function. *Genes & Development*.

